# Pepperberg plot: Modeling flash response saturation in retinal rods of mouse

**DOI:** 10.3389/fnmol.2022.1054449

**Published:** 2023-01-13

**Authors:** Giovanni Caruso, Colin Klaus, Heidi E. Hamm, Vsevolod V. Gurevich, Paolo Bisegna, Daniele Andreucci, Emmanuele DiBenedetto, Clint L. Makino

**Affiliations:** ^1^Italian National Research Council, Istituto di Scienze del Patrimonio Culturale, Rome, Italy; ^2^The College of Public Health Division of Biostatistics and The Mathematical Biosciences Institute, The Ohio State University, Columbus, OH, United States; ^3^Department of Pharmacology, Vanderbilt University Medical Center, Nashville, TN, United States; ^4^Department of Civil Engineering and Computer Science, University of Rome Tor Vergata, Rome, Italy; ^5^Department of Basic and Applied Sciences for Engineering, Sapienza University of Rome, Rome, Italy; ^6^Department of Mathematics, Vanderbilt University, Nashville, TN, United States; ^7^Department of Physiology & Biophysics, Boston University Chobanian & Avedisian School of Medicine, Boston, MA, United States

**Keywords:** visual transduction, RGS9, PDE, CNG channel, membrane guanylate cyclase, cyclic GMP, GRK, G protein

## Abstract

Retinal rods evolved to be able to detect single photons. Despite their exquisite sensitivity, rods operate over many log units of light intensity. Several processes inside photoreceptor cells make this incredible light adaptation possible. Here, we added to our previously developed, fully space resolved biophysical model of rod phototransduction, some of the mechanisms that play significant roles in shaping the rod response under high illumination levels: the function of RGS9 in shutting off G protein transducin, and calcium dependences of the phosphorylation rates of activated rhodopsin, of the binding of cGMP to the light-regulated ion channel, and of two membrane guanylate cyclase activities. A well stirred version of this model captured the responses to bright, saturating flashes in WT and mutant mouse rods and was used to explain “Pepperberg plots,” that graph the time during which the response is saturated against the natural logarithm of flash strength for bright flashes. At the lower end of the range, saturation time increases linearly with the natural logarithm of flash strength. The slope of the relation (τ_D_) is dictated by the time constant of the rate-limiting (slowest) step in the shutoff of the phototransduction cascade, which is the hydrolysis of GTP by transducin. We characterized mathematically the X-intercept (Φo) which is the number of photoisomerizations that just saturates the rod response. It has been observed that for flash strengths exceeding a few thousand photoisomerizations, the curves depart from linearity. Modeling showed that the “upward bend” for very bright flash intensities could be explained by the dynamics of RGS9 complex and further predicted that there would be a plateau at flash strengths giving rise to more than ~10^7^ photoisomerizations due to activation of all available PDE. The model accurately described alterations in saturation behavior of mutant murine rods resulting from transgenic perturbations of the cascade targeting membrane guanylate cyclase activity, and expression levels of GRK, RGS9, and PDE. Experimental results from rods expressing a mutant light-regulated channel purported to lack calmodulin regulation deviated from model predictions, suggesting that there were other factors at play.

## Introduction

Vision in dim light is mediated by rod photoreceptors in the retina that capture single photons and convert them into electrical signals *via* a biochemical cascade (reviewed in Zang and Neuhauss, 2021; Kawamura and Tachibanaki, 2022; Wensel, 2024). Briefly, photoexcited rhodopsin (R^*^) catalyzes activation of the rod-specific G protein transducin (T in the inactive state, T^*^ in the active state), that stimulates a cGMP phosphodiesterase, PDE. The ensuing drop in cGMP levels closes cyclic-nucleotide-gated (CNG) cation channels in the plasma membrane, suppressing an inward current carried by Na^+^ and to a lesser extent, by Ca^2+^. Absorption of a single photon closes 5–10% of the open channels. The response terminates following phosphorylation of R^*^ by rhodopsin kinase (GRK1 or RK) and subsequent arrestin-1 binding and by GTP hydrolysis by T^*^. Membrane guanylate cyclases (ROS-GCs) restore the cGMP levels enabling the CNG channels to reopen.

There is a light-induced fall in intracellular Ca^2+^ because upon closure of CNG channels, the extrusion of Ca^2+^ by a sodium/potassium/calcium exchanger exceeds its influx. The lowered Ca^2+^ stimulates cGMP synthesis by ROS-GCs to quicken the restoration of cGMP and reopen CNG channels, accelerates R^*^ shutoff by causing recoverin to release RK, and increases the affinity of CNG channels for cGMP.

The photocurrent response saturates with a flash that is sufficiently bright to close all CNG channels. Further increases in flash strength prolong the duration of the response. Although rods no longer convey meaningful visual information under bright illumination, studies of the saturation behavior yield important insights into the molecular mechanisms of the phototransduction cascade. David Pepperberg discovered that over a limited range, the time in saturation of a bright flash response increases linearly with the log of the flash strength (Pepperberg et al., 1992). The rationale is that with powerful cascade activation, the cGMP levels drop far below that required to hold open CNG channels. As phototransduction cascade activity declines and cGMP levels recover, the channels reopen along an exponential time course (Pepperberg et al., 1992; Nikonov et al., 2000). The slope of the relation between natural logarithm of the flash strength and time in saturation gives the time constant τ_D_ for the slower of the two steps, rhodopsin quench and shutoff of transducin-activated PDE. The latter was identified as rate-limiting (Krispel et al., 2006). For very bright flashes, the relation curves upward, indicating slowing of cascade shutoff. Here we used mathematical modeling to explain the saturation behavior of WT rods as well as the behaviors of rods of a number of mutant mice that were genetically engineered to modify their phototransduction cascades. To simplify calculations and reduce computation time, we implemented a globally well-stirred (GWS) version of our fully space resolved (FSR) model as described in (Andreucci et al., 2003; Bisegna et al., 2008; Caruso et al., 2011, 2019), because with hundreds of activated rhodopsins randomly distributed among rod discs and with random positions of active rhodopsin within each disc, the two models were equivalent. Modeling efforts were directed to mouse rods, for which many transgenic variants have been described.

## Materials and methods

Simulations of the ordinary differential equation systems in this paper were performed in Matlab. The routines have been made available at https://github.com/klauscj68/Pepperberg.Symbols for the parameters used for mouse rods and their values are given in Supplementary Table S1. Minor adjustments were made within published ranges to optimize the fitting. Pepperberg curves for various mutant mice were taken from studies previously reported in the literature. A collecting area of 0.45 μm^2^ was used to convert flash strength to photoisomerizations (Φ), based on measurements of rod outer segment dimensions (Burns and Pugh, 2009).

## Results and discussion

### Model of phototransduction

Intracellular Ca^2+^ drops significantly in the ROS during saturating responses, impacting a number of cascade reactions. Our FSR model was therefore further developed to include two different calcium-dependent membrane guanylate cyclase activities, calcium-dependent modulation of the CNG channel, and calcium-dependent phosphorylation of R^*^, as described below. In addition, the GWS version of the FSR model incorporated regulation of the shutoff of T^*^-activated PDE activity by RGS9.

#### Second messengers of the phototransduction cascade

The rate equations for cGMP and Ca^2+^ are:


(1a) $$\begin{array}{c} \begin{array}{l} \frac{d}{dt}\left[cGMP\right]=\alpha_{\min}+\left(\alpha_{\max}-\alpha_{\min}\right)\\ \;\;\;\;\;\;\;\;\;\left[\frac{\beta }{1+{\left(\left[Ca^{2+}\right]/K_{cyc1}\right)}^{m_{cyc1}}}+\frac{1-\beta }{1+{\left(\left[Ca^{2+}\right]/K_{cyc2}\right)}^{m_{cyc2}}}\right]\\ \; \end{array}\\ \;-k_{hyd}\left(E_{tot}-E^{*}\right)\left[cGMP\right]-k_{\sigma ;hyd}E^{*}\left[cGMP\right], \end{array}$$

(1b) $$\frac{d}{dt}\left[Ca^{2+}\right]=\eta \left(\frac{1}{2}f_{Ca}J_{cG}-J_{ex}\right)$$

where $\beta \in \left[0,1\right],$


(2b) $$\begin{array}{c} J_{cG}=\frac{j_{cG}^{\max}}{V_{cyt}}\frac{{\left[cGMP\right]}^{m_{cG}}}{K_{cG}{}^{m_{cG}}+{\left[cGMP\right]}^{m_{cG}}} \end{array},$$

(2b) $$\begin{array}{c} J_{ex}=\frac{j_{ex}^{sat}}{V_{cyt}}\frac{\left[Ca^{2+}\right]}{K_{ex}+\left[Ca^{2+}\right]}. \end{array}$$

In mouse rod outer segments, cGMP is synthesized by two membrane guanylate cyclases, ROS-GC1 and ROS-GC2, that are regulated by two neuronal Ca^2+^ sensors, GCAP1 and GCAP2, with different affinities for Ca^2+^ (reviewed in Wen et al., 2014). Their activities are described by the terms in brackets on the right-hand side of eq. (1a). The constants $\alpha_{\min}<\alpha_{\max}$, in $\mu M\;s^{-1}$ are given by the production rates of cGMP at maximum and minimum Ca^2+^ concentration (theoretically as $\left[Ca^{2+}\right]\to \infty$ and $\left[Ca^{2+}\right]\to 0,$ respectively). $K_{cyc}$ in μM is the Ca^2+^ concentration for which the rate of production of cGMP by a ROS-GC is half-maximal. The number $m_{cyc}$ is the dimensionless Hill’s exponent. ROS-GC2 pairs primarily with GCAP2 while ROS-GC1 pairs with either GCAP, but for modeling purposes, it is only important to distinguish the overall Ca^2+^ dependent activities. In the last two terms, $k_{hyd}$ and $k_{\sigma ;hyd}$ in μm^3^ $s^{-1}$ are the catalytic rates of hydrolysis of cGMP by basal and activated PDE subunits, respectively.

The term $J_{cG}$ is the current carried by the CNG channels. The multiplying constant $f_{Ca}$ is the dimensionless fraction of cGMP-activated current carried by Ca^2+^. The constant $j_{cG}^{\max}$, in pA, is the current for maximal [cGMP] (theoretically as [cGMP]→∞), whereas $K_{cG}$, in μM is the cGMP concentration corresponding to half-maximal channel opening, and $m_{cG}$ is the dimensionless Hill’s exponent. The parameter $K_{cG}$ is itself a function of Ca^2+^ due to the presence of calmodulin as described by the equation:


(3) $$\begin{array}{c} \begin{array}{l} K_{cG}\left(\left[Ca^{2+}\right]\right)=K_{cG,\max}+\left(K_{cG,\min}-K_{cG,\max}\right)\\ \;\;\;\;\;\;\;\;\;\;\;\;\;\;\;\;\;\;\;\;\;\;\;\;\;\;\;\;\;\;\;\;\;\;\;\;\;\;\;\;\;\;\;\;\;\;\;\frac{K_{CaM}^{m_{CaM}}}{K_{CaM}^{m_{CaM}}+{\left[Ca^{2+}\right]}^{m_{CaM}}} \end{array} \end{array}$$

where $m_{CaM}$ is the Hill’s exponent for calmodulin activation by Ca^2+^, the constant $K_{CaM}$ is the Ca^2+^ concentration for a half-maximal calmodulin effect and $K_{cG,\min}$ and $K_{cG,\max}$ are the minimum and maximum binding affinities of each of the cGMP binding sites on the channel, occurring for $\left[Ca^{2+}\right]\to 0$ and $\left[Ca^{2+}\right]\to \infty$ respectively.

The term $J_{ex}$ is the current due to Na^+^/Ca^2+^, K^+^ exchange, $j_{ex}^{sat}$ in pA is the saturated exchange current occurring at maximum $\left[Ca^{2+}\right]$ (theoretically for $\left[Ca^{2+}\right]\to \infty$) and $K_{ex}$ in μM is the [Ca^2+^] at which the exchange current is half-maximal. The constant η is of the form ${\left(B_{Ca}F\right)}^{-1}$, where $B_{Ca}$ is a dimensionless constant that takes into account the Ca^2+^-buffering effects in the cytosol and F=96,500 C mol^−1^ is the Faraday constant. With these specifications, the units in eq. (1b) are self-consistent.

#### Dynamics of R* shutoff

Photon absorption converts R to R^*^, which is a substrate for phosphorylation by RK. Phosphorylation decreases the ability of R^*^ to activate transducin, but complete quench only occurs after the binding of arrestin-1 (Arr). Denote by $\left[R_{j}^{*}\right]$ the concentrations of activated rhodopsins in their j^th^ phosphorylated state, identified with the number j−1 phosphates attached to $R^{*}$ up to a maximum of six phosphorylations (for mouse). Newly activated $R^{*}$ molecules have no attached phosphates, so they are in the state j=1. Rhodopsins in the state j transition either to the state j+1 at a rate of $\lambda_{j}$ or they bind to Arr at a rate $\mu_{j}$, thereby being quenched. Removal of phosphates from R^*^ occurs on a much slower time scale (Berry et al., 2016) and is disregarded in the model. The quantities $\lambda_{j}$ and $\mu_{j}$ measure, in $s^{-1}$, the binding rates of $R_{j}^{*}$ to RK and Arr, respectively. The numbers ${\left(\lambda_{j}+\mu_{j}\right)}^{-1}$ measure the statistical average of the sojourn time of an activated rhodopsin in the j^th^ state. It is assumed that $\mu_{j}$ of Arr to $R_{j}^{*}$ depends only on the j^th^ phosphorylated state. For example, *μ*_*j*_ is zero if all phosphorylated states are removed or prevented, e.g., by knockout of RK. For the model, all $\mu_{j}$ values were set to zero for j < 4 and set to the maximal rate of arrestin binding, $\mu_{\max}$, for j ≥ 4. Similarly, $\lambda_{n+1}=0$ after $R_{n+1}^{*}$ binds Arr (is quenched). The quantities $\lambda_{j}$ are functions of any biochemical process that either increases or decreases the probability of $R_{j}^{*}$ binding RK. For example, a $\left[Ca^{2+}\right]$ drop releases RK from its complex with recoverin, thereby increasing the probability of $R^{*}$ binding RK and thus shortening the sojourn times before the next phosphorylation. While other dependencies might be present, we assume that $\lambda_{j}=\lambda_{j}\left(\left[Ca^{2+}\right]\right)$ where the form of this function has to be specified. For j≥2, the $\left[R_{j}^{*}\right]$ is augmented by $\lambda_{j-1}\left[R_{j-1}^{*}\right]$, that is the $\left[R_{j}^{*}\right]$ imported from the previous state, and is depleted by $\left(\lambda_{j}+\mu_{j}\right)\left[R_{j}^{*}\right]$, that is by the $\left[R_{j}^{*}\right]$ that transitions to the next state or is quenched. Thus, flashes produce only new [$R_{1}^{*}]$, which is depleted by $\left(\lambda_{1}+\mu_{1}\right)\left[R_{1}^{*}\right]$, and localized at times of flashes by the Dirac mass $\delta_{t}$. The rate equations for [$R_{j}^{*}$] are:


(4a) $$\begin{array}{c} \;\frac{d}{dt}\left[R_{1}^{*}\right]=\Phi \delta_{t}-\left(\lambda_{1}+\mu_{1}\right)\left[R_{1}^{*}\right] \end{array},$$

(4b) $$\begin{array}{c} \frac{d}{dt}\left[R_{j}^{*}\right]=\lambda_{j-1}\left[R_{j-1}^{*}\right]-\left(\lambda_{j}+\mu_{j}\right)\left[R_{j}^{*}\right]\;\mathrm{for}\;j=2,\ldots ,n. \end{array}$$

The [*RK*] and hence, the phosphorylation rates of the activated rhodopsin are a function of the $\left[Ca^{2+}\right]$ that changes over the course of a flash response. A description for the case of single step rhodopsin quenching and well stirred conditions will be adopted here for sequential, multiple phosphorylations and final quenching. Only the phosphorylation rates are influenced by the calcium concentration drop, whereas Arr binding rate is assumed to be calcium-independent. By extending equation (A12) of Nikonov et al. (2000):


(5) $$\begin{array}{c} \frac{\lambda_{i}\left(\left[Ca^{2+}\right]\right)}{\lambda_{i,\max}}=\frac{\left[RK\right]}{{\left[RK\right]}_{tot}}. \end{array}$$

This equation holds true for a single-step deactivation, for which $k_{R}=\lambda$ and $k_{R,\max}=\lambda_{\max}$. Thus, it is assumed that the same biochemical relationship holds at each state of $R^{*}$. From equation (C1) of Nikonov et al. (2000):


(6) $$\begin{array}{c} \frac{\left[RK\right]}{{\left[RK\right]}_{tot}}={\left(1+C_{1}\frac{\left[Rec\right]}{{\left[Rec\right]}_{tot}}\right)}^{-1}. \end{array}\;$$

According to equations (C2) and (C4) of Nikonov et al. (2000):


(7a) $$\begin{array}{c} C_{1}={\left(\frac{\left[Ca^{2+}\right]}{K_{1}}\right)}^{2}\left[\frac{1}{K_{3}}+\frac{1}{K_{4}}\frac{M}{K_{2}}\right]{\left[Rec\right]}_{tot} \end{array},$$

(7b) $$\begin{array}{c} C_{2}=1+{\left(\frac{\left[Ca^{2+}\right]}{K_{1}}\right)}^{2}\left(1+\frac{M}{K_{2}}\right). \end{array}$$

Then the dependence on $\left[Ca^{2+}\right]$ comes from the algebraic second order equation (C3) of Nikonov et al. (2000):


(8) $$\begin{array}{c} C_{1}C_{2}{\left(\frac{\left[Rec\right]}{{\left[Rec\right]}_{tot}}\right)}^{2}+\left[C_{1}\left(\frac{{\left[RK\right]}_{tot}}{{\left[Rec\right]}_{tot}}-1\right)+C_{2}\right]\frac{\left[Rec\right]}{{\left[Rec\right]}_{tot}}-1=0. \end{array}$$

Here $K_{i}$ for i=1,…,4 and M are biochemical parameters defined in Nikonov et al. (2000).

#### Activation/deactivation of transducin and PDE

During its average lifetime $\tau_{R^{*}}$, photoexcited rhodopsin R^*^ activates many transducins ($T\to T^{*}$) by catalyzing GTP exchange for the GDP bound to their α-subunits. Each molecule of T^*^ associates, one-to-one, with a catalytic subunit of the effector forming a T^*^--E complex. Full activation of the catalytic subunit is assumed, denoted by E^*^. A single molecule of E^*^, during its average lifetime $\tau_{E^{*}}$, hydrolyzes over 50 molecules of the second messenger cGMP which, dissociating from the cationic channels they keep open, causes channel closure and thereby suppression of the inward current $J_{cG}$ (Pugh and Lamb, 1993 2000). T^*^ possesses an intrinsic GTPase activity that terminates T^*^--E activity. The synthesis of cGMP overtakes hydrolysis and as the levels rise, CNG channels reopen, ending the time in saturation, $T_{sat}$.

Let [*T*]_0_ and [T^*^]denote the initial, basal concentration of transducin in the rod outer segment (ROS) and the concentration of activated transducin, respectively. Also, let [*E*]_0_ and [E^*^] denote the concentrations of subunits of the effector PDE in darkness and of subunits of activated effector PDE^*^, respectively. Assuming independent activation of each PDE subunit, ${\left[PDE\right]}_{0}=\frac{1}{2}{\left[E\right]}_{0}$ and$\;\left[PDE^{*}\right]=\frac{1}{2}\left[E^{*}\right],$ the rate equations for [T^*^] and [E^*^], for a well-stirred activation/deactivation model are:


(9a) $$\begin{array}{c} \frac{d}{dt}\left[T^{*}\right]=\left(\frac{{\left[T\right]}_{0}-\left[T^{*}\right]}{{\left[T\right]}_{0}}\right)\sum_{j=1}^{n}\nu_{j}\left[R_{j}^{*}\right]-k_{T^{*}E}\left({\left[E\right]}_{0}-\left[E^{*}\right]\right)\left[T^{*}\right] \end{array}$$

(9b) $$\begin{array}{c} \frac{d}{dt}\left[E^{*}\right]=k_{T^{*}E}\left({\left[E\right]}_{0}-\left[E^{*}\right]\right)\left[T^{*}\right]-k_{E}\left[E^{*}\right]. \end{array}$$

The constant $k_{T^{*}E}$ in μm^2^ $s^{-1}$, is the rate of formation of the complex T^*^--E. It is assumed that activation of a molecule of transducin occurs upon encounter with a molecule of activated rhodopsin. Thus, the first term on the right-hand side of eq. (9a) measures the increase of $\left[T^{*}\right]$ due to activated rhodopsin [$R^{*}]$. The second term measures its depletion due to its binding to PDE. The constant $k_{E^{*}}$ = 1/τ_E_, where τ_E_ is the average E^*^ lifetime dictated by the deactivation rate of the T^*^--E complex. It is remarked that eqs. (9a, b) represent a simplified model of the disc cascade widely used in the literature. In order to study the behavior of rod cells under a high saturating illumination, a more detailed model is proposed and used for the numerical simulations, as reported in eqs. (21a-f), explicitly accounting for the RGS9 dynamics.

#### Derivation of the Pepperberg equation

**Figure 1:**
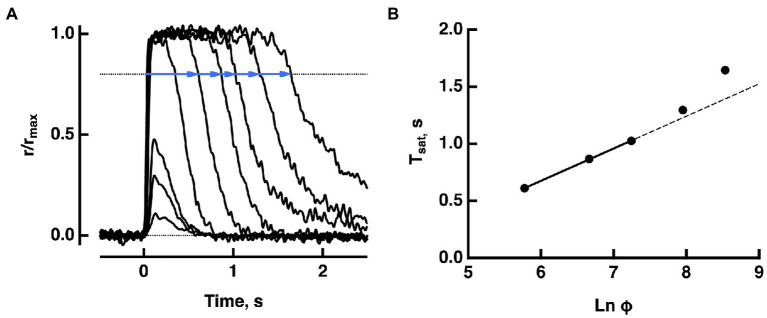
Pepperberg plot for a WT mouse rod. **(A)** Photocurrent responses of a rod to flashes of increasing strength, presented at time zero. For the brightest 5 flashes, saturation time (*T*_*sat*_) was measured from mid-flash to a criterion recovery of 20% (blue lines). **(B)** Pepperberg plot. Flash strength was converted to photoisomerizations (Φ), using a collecting area of 0.45 μm^2^. The three shortest saturation times were fitted with a line; longer saturation times deviated from the line. The slope of the line τ_D_ = 283 ms, provided an estimate of τ_E*,_ the time constant for the shutoff of T^*^——E. Adapted from Makino et al. (2008).

**Figure 2:**
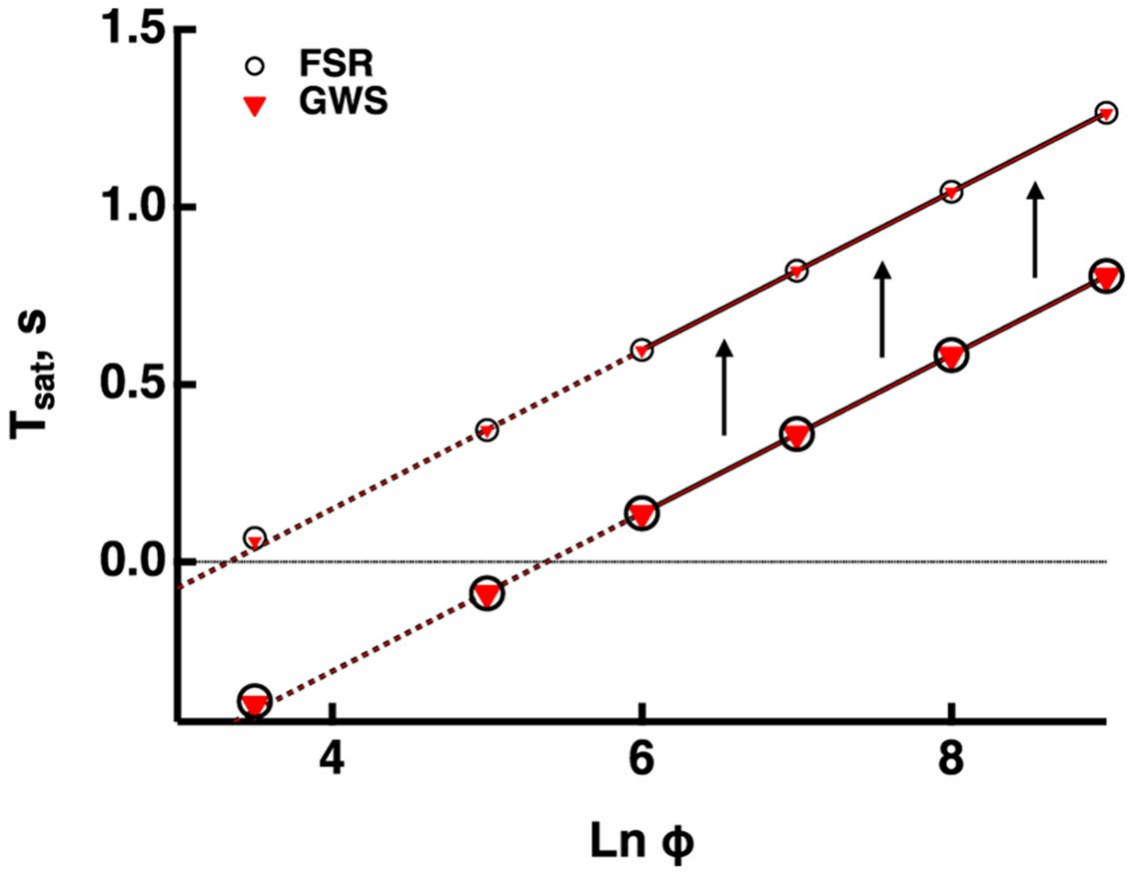
Validation of the GWS version of the model. GWS (red triangles) and FSR (open circles) models yielded nearly identical responses to bright flashes in a mouse rod. Only the four brightest flashes were saturating. The lines passing through the associated saturation times was extended to the left with dashed lines. Saturation time, *T*_*sat*_, was measured from mid-flash to 2% recovery (larger symbols). For the purposes of illustration, *T*_*sat*_ was measured in the same way for the subsaturated responses to the two weakest flashes. For comparative purposes, *T*_*sat*_ was also measured from mid-flash to 20% recovery (smaller symbols). The slope of the Pepperberg relation was unaffected by the change in criterion recovery, but there was an upward translation equivalent to a lateral shift to a lower X-intercept.

Suppose that a bright flash Φ (in instantaneous number of isomerizations) applied at time t=0 causes cGMP levels to fall below the minimum required for CNG channel opening. Then the output current drop saturates for a time, $T_{sat}$ (Figure 1). In practice, there are technical difficulties in measuring $T_{sat}$; bright flash responses do not have sharp transitions into or out of saturation on the rising and recovery phases. Although $J_{cG}$ terminates abruptly, the decline in $J_{ex}$ carried by the sodium/potassium/calcium exchanger follows a slower time course and there is noise that is omnipresent in electrical recordings. To circumvent these difficulties, saturation time is typically measured from mid-flash or from flash onset to a criterion recovery of the response. Such a practice inflates $T_{sat}$, but the upward shift does not alter the slope of the linear portion of the Pepperberg curve (e.g., Figure 2). Various publications have adopted different criterion recovery levels, so care must be taken in comparing the X-intercepts across studies.

$T_{sat}$ increases linearly with $Ln\left(\Phi \right)$, with slope τ_D_ that is determined by the shutoffs of R^*^ and/or T^*^--E (Pepperberg et al., 1992, 1994). It was shown that the shutoff of T^*^--E was slower than that of R^*^ (Krispel et al., 2006), so assuming that $\tau_{R^{*}}\;<<\tau_{E^{*}}$, the value of $\tau_{E^{*}}$ can be determined from the slope of the Pepperberg curve. Hence,


(10) $$T_{sat}=\tau_{E^{*}}Ln\left(\Phi \right)+C$$

for a constant *C* > 0 that has not been described previously and will be discussed in further detail below.

In deriving the Pepperberg curve equation, a well stirred condition was assumed, i.e., all concentrations were taken to be uniform inside the ROS. Moreover, it was assumed that the rhodopsins were all of one species denoted by R^*^. With this stipulation only eq. (4a) was in force and


(11) $$\frac{1}{\left(\lambda_{1}+\mu_{1}\right)}=\frac{1}{\lambda }=\tau_{R^{*}}$$

the average lifetime of R^*^. For a saturating flash Ф applied to the system at time *t* = 0,


(12) $$\left[R^{*}\right]=\Phi e^{-t/\tau_{R^{*}}}\;\mathrm{for}\;t>0.$$

It was further assumed that the effects of the intermediate activation of transducin can be lumped into a direct activation of E^*^ by R^*^, then by a modified form of eq. (9b):


(13) $$\begin{array}{c} \frac{d}{dt}\left[E^{*}\right]=\nu_{RE}\left[R^{*}\right]-k_{E}\left[E^{*}\right]\;\mathrm{with}\;\left[E^{*}\right]\left(0\right)=0. \end{array}$$

Integrating over $\left(0,T_{sat}\right)$ where $T_{sat}$ is a positive number to be defined:


(14) $$\begin{array}{c} {}[E^{*}]\left(T_{sat}\right)=\nu_{RE}\Phi \frac{\tau_{R^{*}}\tau_{E^{*}}}{\tau_{E^{*}}-\tau_{R^{*}}}\left(e^{-T_{sat}/\tau_{E^{*}}}-e^{-T_{sat}/\tau_{R^{*}}}\right)\\ \approx \Phi \nu_{RE}\tau_{R^{*}}e^{-T_{sat}/\tau_{E^{*}}} \end{array}$$

for $\tau_{R^{*}}<<\tau_{E^{*}}$. From this:


(15) $$\begin{array}{c} T_{sat}\approx \tau_{E^{*}}Ln\left(\Phi \right)+\tau_{E^{*}}Ln\left(\frac{\nu_{RE}\tau_{R^{*}}}{\left[E^{*}\right]\left(T_{sat}\right)}\right). \end{array}$$

We notate $E_{char}^{*}=[E^{*}](T_{sat})$. It is a fundamental quantity that characterizes an essential feature of the system. To define $T_{sat}$ and compute $E_{char}^{*}$, refer back to eq. (1a), viewed at times of saturation, originating from the dark-adapted state. Stipulate that the interval (0, $T_{sat}$) lies within the saturation regime, and that in such a regime, [*cGMP*] and [*Ca*^2+^] are in quasi steady state. Thus, we may set approximately to zero the time-derivative in eqs. (1a) and (1b). Since $k_{hyd}<<k_{\sigma ,hyd}$, the term $k_{hyd}E^{*}\left[cGMP\right]$ can be neglected. It then follows from eqs. (1a) and (1b):


(16a) $$\alpha_{\max}-k_{hyd}E_{tot}\left[cGMP\right]-k_{\sigma ;hyd}E_{char}^{*}\left[cGMP\right]=0,$$

(16b) $$\frac{1}{2}f_{Ca}J_{cG}-J_{ex}=0.$$

This relation holds for all times during which saturation is in force. For the same times, eq. (16b) yields $J_{ex}=\frac{1}{2}f_{Ca}J_{cG}$. Therefore, the total photocurrent is


(17) $$\begin{array}{c} J=J_{cG}+J_{ex}=\left(1+\frac{1}{2}f_{Ca}\right)J_{cG}. \end{array}$$

Let $J_{\max}$ be the maximum current that the ROS can output (typically, $J_{\max}=J_{dark})$. Then define $T_{sat}$ as the time for which


(18) $$\begin{array}{c} J\left(T_{sat}\right)=\varepsilon J_{\max},\;\;for\;a\;fixedsmall\;\varepsilon \in \left(0,1\right). \end{array}$$

Eqs. (16b) and (18) can be solved for the unknowns [*cGMP*] and [*Ca*^2+^] at time $T_{sat}$. This permits the determination of $E_{char}^{*}$ by eq. (16a). Inserting it in eq. (15) gives a linear relation between $T_{sat}$ and $Ln\left(\Phi \right)$, with slope $\tau_{E^{*}}$, of the form


(19) $$\begin{array}{c} \begin{array}{l} T_{sat}=\tau_{E^{*}}Ln\left(\Phi \right)-\tau_{E^{*}}Ln\left(\Phi o\right)\\ \mathrm{where}\Phi o=\frac{E_{char}^{*}}{\nu_{RE}\tau_{R^{*}}}. \end{array} \end{array}$$

The quantity Φo can be identified as the flash strength for which $T_{sat}=0$. Measurement of Φo could permit the determination of $E_{char}^{*}$.

The constant *C* in eq. (10), is mathematically derived above as


(20) $$\begin{array}{c} C=-\tau_{E^{*}}Ln\left(\Phi o\right) \end{array}.$$

It is emphasized that *C* is proportional to $\tau_{E^{*}}$ and independent of Φ. This form of *C* implies that the straight lines described by eq. (19) corresponding to different values of $\tau_{E^{*}}$ have a common point (i.e., Φ=Φoand $T_{sat}=0$) regardless of their slope. This feature actually occurs in the experimental curves of Krispel et al. (2006), reported in (Burns and Pugh, 2009) and is discussed below.

### Modeling the Pepperberg plots of mutant mouse rods

#### Testing the validity of the GWS model

Outputs from the FSR model and the GWS version converge at very bright flashes. Since usage of the GWS version greatly facilitates numerical simulations, we ascertained whether it would be adequate for responses even to the weakest saturating flashes by computing bright flash responses for mouse rods using both models with the parameter set for mouse in the Supplementary Table S1. Figure 2 compares the Pepperberg curves; the close match of the curves verifies that even for two flash strengths that do not quite saturate the response, any spatial inhomogeneity of cascade activity was not distinguished by the two versions of the model. Hereafter, all simulations were made using the GWS model.

Since experimentally determined values for $T_{sat}$are always measured after some arbitrary criterion recovery, they are inflated relative to the true saturation times and the X-intercept is shifted to the left, underestimating the true values for Φo and E^*^_char_ that describe the minimal conditions for saturation (see above). For modeled responses in Figure 2, we calculated that the $T_{sat}$ values determined at 20% recovery were increased by 0.46 ms, compared to those determined at 2% recovery and that this had the effect of translating the X-intercept along the abscissa from a $Ln\left(\Phi \right)$ of 3.3 to 5.3, i.e., by a factor of 7.4-fold. Such a correction could be applied to the Pepperberg plots in the figures below for which $T_{sat}$was measured from mid-flash to 20% recovery, to improve the estimates of Φo and E^*^_char_. The magnitude of the translation would have to be adjusted for determinations of $T_{sat}$ made at different criterion levels of response recovery.

#### Upward bend in the Pepperberg curve due to depletion of the RGS9 complex

**Figure 3:**
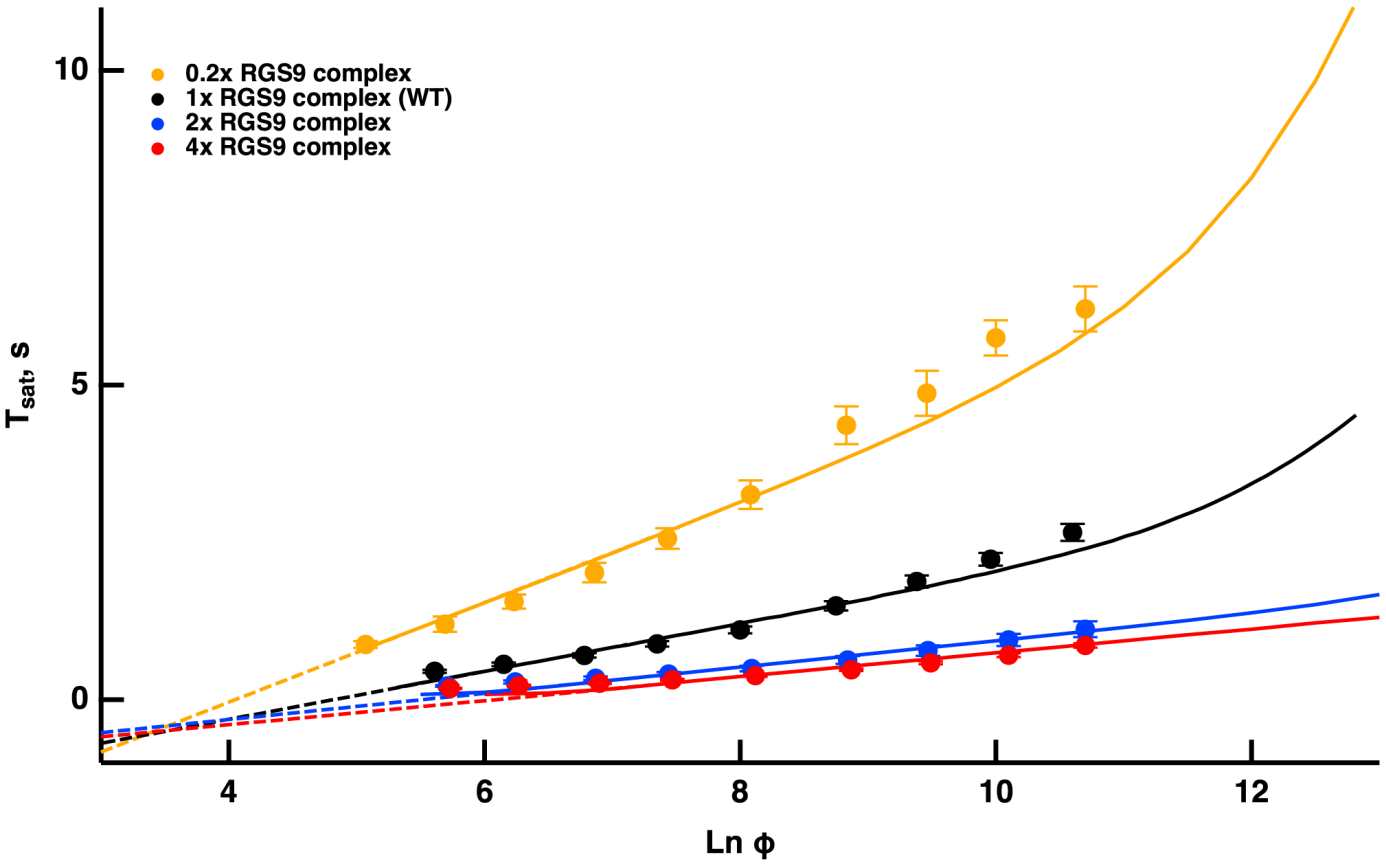
Fits of the model to experimental results of mouse rods with different expression levels of the RGS9 complex. For modeling, levels of RGS9 complex in the mutant rods were changed to 0.2x, 2x and 4x that of WT. Experimental results for R9AP+/−, R9AP75 and R9AP138 lines of mice were from Burns and Pugh (2009). *T*_*sat*_ was measured from mid-flash to 10% recovery. Continuous lines show the fits with the model, where the linear portions of the relations were extended with dashed lines to show that they intersect near *T*_*sat*_ = 0.

Once activated, T relieves an autoinhibition of PDE, allowing it to hydrolyze cGMP. The GTPase activity of T^*^ terminates the interaction between T^*^ and PDE. The intrinsic GTPase activity is slow, but is greatly enhanced by the encounter with an RGS9 complex. By showing that an increase in the expression level of the RGS9 complex accelerated flash response recovery and reduced the slope of the Pepperberg relations in rods, Krispel et al. (2006) validated the idea that the collisional delay of RGS9 complex with T^*^--E was the major determinant of T^*^ lifetime and identified the shutoff of T^*^--E as the rate-limiting step in the response recovery. Attempts to accelerate R^*^ shutoff by overexpression of RK did not alter the slope of the Pepperberg plot. Our model captured the Pepperberg relations for the four types of rods described in Burns and Pugh (2009), each expressing a different level of RGS9 (Figure 3). The values for $\tau_{E^{*}}$ from our model were: 792 ms for rods expressing 0.2x the normal amount of RGS9 complex, 381 ms for WT, 209 ms for 2-fold overexpression, and 191 ms for 4-fold overexpression. The values differ somewhat from the population averages of: 246 ms for WT, 108 ms for 2-fold overexpression, 80 ms for 4-fold overexpression obtained from linear fits over the lower range of flash strengths (Krispel et al., 2006) but conserve the overall trend.

**Figure 4:**
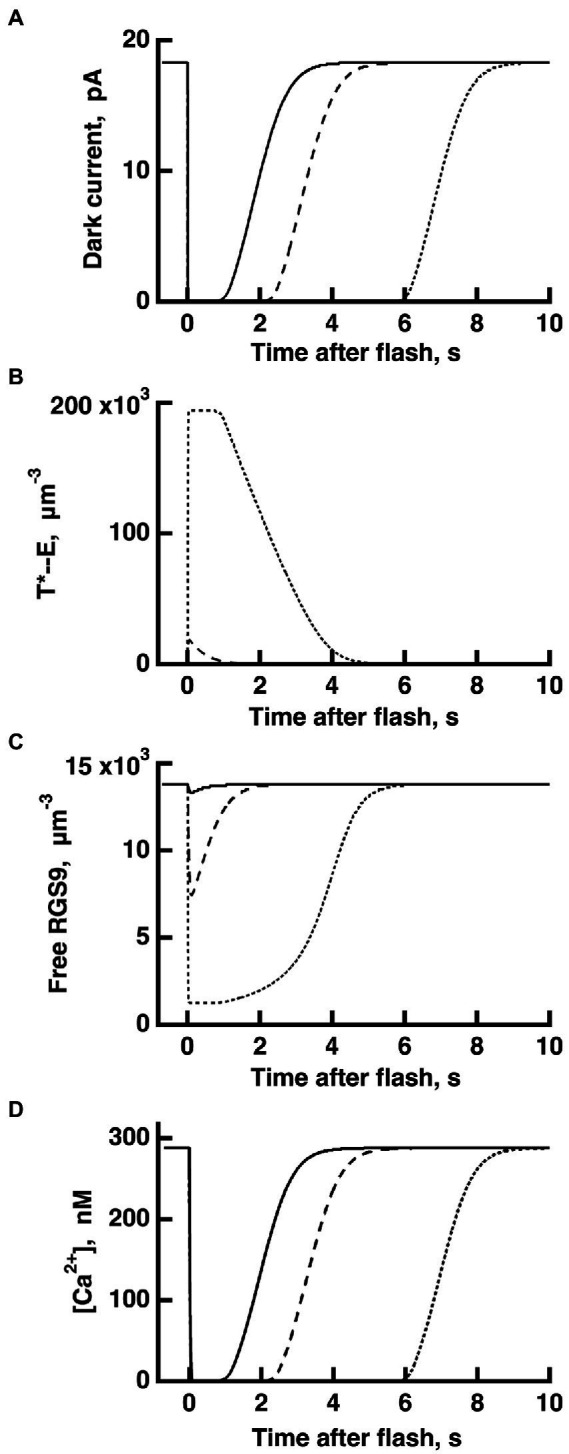
WT rod simulations for three flash responses **(A)**, and the associated dynamics for T^*^——E **(B)**, free RGS9 **(C)** and Ca^2+^ **(D)**. The flash was given at time zero. The three flashes elicited responses with *T*_*sat*_ values in the initial, linear segment of the Pepperberg plot, near the transition to the upward bend, and well within the upward bend, respectively (*cf.* Figure 3): *Ln* (Φ) = 8 (continuous lines), *Ln* (Φ) = 11 (dashed lines), *Ln* (Φ) = 14 (dotted lines). Dynamics of free RGS9 deviated markedly from that of T^*^——E, and greatly prolonged *T*_*sat*_ for the response to the brightest flash.

Since the timing of T^*^ shutoff (i.e., $\tau_{E^{*}}$) is too slow to affect the rising phase of the bright flash response, rods of each expression level should have reached saturation at a similar flash strength and all Pepperberg curves should have converged at the same X-intercept, Φo_._ The curves nearly converged, although the intersection fell slightly below $T_{sat}$ = 0. We attribute the deviations of the experimental results from the model to biological variation and to technical issues associated with achieving the same collecting area for every rod during recording.

Burns and Pugh (2009) proposed a model for the experimentally obtained Pepperberg results of rods expressing various levels of RGS9 complex that consists essentially of eq. (9b), where $k_{E}$ is replaced by $k_{f}\left[RGS9\right]$ for a suitable constant $k_{f}$ to be computed by fitting. Since $\tau_{E^{*}}$ is the reciprocal of $k_{E}$, the larger [*RGS9*] the smaller $\tau_{E^{*}}$ and vice versa. A key assumption of that model is that [*RGS9*] remains *constant* over the range of light intensities that they analyzed. While the fit is good for lower saturating flash intensities (less than ~3,000 photoisomerizations), saturation times depart from the curves at higher flash intensities, becoming non-linear and exhibiting an upward, convex, super-linear bending (Figure 3). It would appear that a new mechanism dominates the response recovery at these flash strengths.

Because the convexity became more prominent in rods underexpressing the RGS9 complex, and was absent from rods overexpressing it, we propose that [*RGS9*] does not remain constant throughout the process. While the total mass ${\left[RGS9\right]}_{0}$ is constant, it dynamically subdivides into a portion [RGS9--T^*^--E] bound to the complex T^*^--E and a portion [*RGS9*_0_] − [RGS9--T^*^--E] available for binding to T^*^--E according to the rate equations:


(21a) $$\begin{array}{c} \frac{d}{dt}\left[T\right]=-\nu_{RT}\left[R^{*}\right]\frac{\left[T\right]}{{\left[T\right]}_{0}}+k_{cat}\left[RGS9--T^{*}--E\right] \end{array},$$

(21b) $$\begin{array}{c} \;\frac{d}{dt}\left[T^{*}\right]=\nu_{RT}\left[R^{*}\right]\frac{\left[T\right]}{{\left[T\right]}_{0}}-k_{T^{*}E}\left[E\right]\left[T^{*}\right] \end{array},$$

(21c) $$\frac{d}{dt}\left[E\right]=-k_{T^{*}E}\left[E\right]\left[T^{*}\right]+k_{cat}\left[RGS9--T^{*}--E\right],$$

(21d) $$\begin{array}{c} \frac{d}{dt}\left[T^{*}--E\right]=k_{T^{*}E}\left[E\right]\left[T^{*}\right]-k_{f}\left[RGS9\right]\left[T^{*}--E\right]\\ +k_{b}\left[RGS9--T^{*}--E\right], \end{array}$$

(21e) $$\begin{array}{c} \frac{d}{dt}\left[RGS9\right]=k_{cat}\left[RGS9--T^{*}--E\right]+k_{b}\left[RGS9--T^{*}--E\right]\\ -k_{f}\left[RGS9\right]\left[T^{*}--E\right], \end{array}$$

(21f) $$\begin{array}{c} \frac{d}{dt}\left[RGS9--T^{*}--E\right]=k_{f}\left[RGS9\right]\left[T^{*}--E\right]\\ -k_{b}\left[RGS9--T^{*}--E\right]\\ -k_{cat}\left[RGS9--T^{*}--E\right], \end{array}$$

with initial conditions


$$\left[T\right]\left(0\right)={\left[T\right]}_{0},\left[E\right]\left(0\right)={\left[E\right]}_{0},\;\;\left[RGS9\right]\left(0\right)={\left[RGS9\right]}_{0},$$

$$\;\left[T^{*}\right]\left(0\right)=\left[T^{*}--E\right]\left(0\right)=\left[RGS9--T^{*}--E\right]\left(0\right)=0$$

where ${\left[T\right]}_{0},{\left[E\right]}_{0},\mathrm{and}\;{\left[RGS9\right]}_{0}$ are the initial, basal concentrations of the transducer G protein, the effector PDE and GTPase-activating protein RGS9. Here $\nu_{RT}$ is the catalytic activity of R^*^ to T^*^, and $k_{T^{*}E}$ is the coupling coefficient between T^*^ and E^*^. Also $k_{f}$ (forward) is the rate of association of the complex $T^{*}--E$per unit mass with available RGS9, and $k_{b}$ (backward) is the rate of dissociation of RGS9 from the complex T^*^--E. Finally, $k_{cat}$ is the rate of deactivation of the RGS9--T^*^--E complex by the hydrolysis of GTP by T^*^.

The dynamics of RGS9 along its time evolution after the flash was traced from its basal values and was observed to lag slightly, the dynamics of T^*^--E for flash strengths $Ln\left(\Phi \right)<8$ (Figure 4). However, for brighter flashes there is an upward, convex, super-linear bending of the Pepperberg relations that the model attributed to a reduction in RGS9 complex availability. According to the model, T^*^ shutoff was delayed by the extra time needed for an RGS9 complex to collide with T^*^--E under these conditions and in the extreme case, would theoretically approach a regime in which shutoff relied on transducin’s intrinsic GTPase activity, unassisted by RGS9.

Reducing $k_{b}$ slows the dissociation of RGS9 from the complex RGS9--T^*^--E and hence, for flashes of equal intensity, the time in saturation$\;T_{sat}$ was shorter. Increasing $k_{f}$ sped up the formation of the complex RGS9--T^*^--E, accelerating the shutoff of T^*^--E, and hence, for flashes of equal intensity, $T_{sat}$ was shorter. Opposite effects occurred by increasing $k_{b}$ or by decreasing $k_{f}$. In all cases, for $Ln\left(\Phi \right)<8$, their behavior is essentially that of a straight line, and in all cases, one observes a convex, superlinear bending with brighter flashes. To provide further evidence that such a pattern is due to RGS9 depletion, the red curve in Figure 3 was obtained by artificially augmenting the basal value [RGS9]_0_ by a factor of 4 and at the same time by reducing the “forward” parameter $k_{f}$ by the same factor. This way, at incipient phases of the process (t≈0), when only a negligible fraction of [*RGS9*]_0_ has been turned into [RGS9--T^*^--E], the product


$$0.25\;k_{f}\left(4{\left[RGS9\right]}_{0}-\left[RGS9--T^{*}--E\right]\right)\approx k_{f}{\left[RGS9\right]}_{0}$$

remains essentially unchanged. Therefore, for low values of [RGS9--T^*^--E] the complex [T^*^--E] dissociates and the complex [RGS9--T^*^--E] is generated at the same rates as in the blue curve in Figure 3. A departure occurs at later times as [*RGS9*]_0_ is depleted, by increasing [RGS9--T^*^--E].

#### Plateau in the Pepperberg curve upon exhaustion of PDE

**Figure 5:**
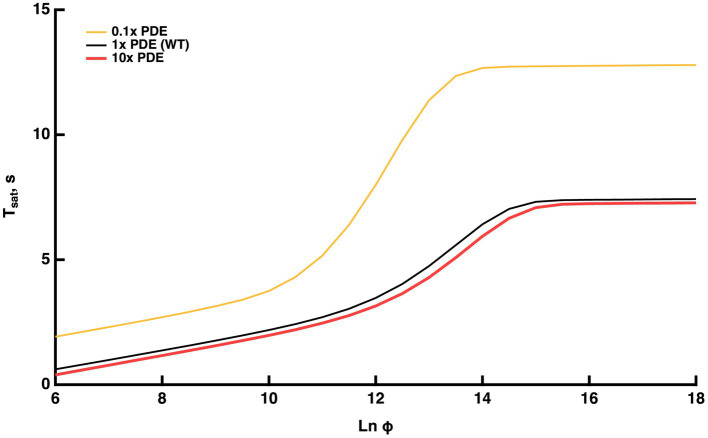
Model prediction that the Pepperberg relations will plateau with flash strengths that fully activate PDE. Predictions are also shown for theoretical variations in the level of PDE expression: 0.1x (10-fold lower level of PDE present than in WT), WT, 10x (10-fold higher level of PDE present than in WT).

For even brighter flashes, $Ln\left(\Phi \right)>15,$, the model predicted an asymptotic flattening in the Pepperberg curve caused by activation of all available PDE (Figure 5). Full activation of PDE achieves the greatest reduction of cGMP, hence it sets an upward limit on $T_{sat}$. To further explore the role of PDE expression, theoretical changes in PDE level were imposed on the model. A tenfold decrement in PDE content actually prolonged $T_{sat}$ at all flash strengths, because there was a reduction in basal PDE activity that elevated cGMP level in darkness, extending the time required for E^*^ activity to return to the dark level after a bright flash (gold trace, Figure 5, explained further in Nikonov et al., 2000 and commentary by Govardovskii et al., 2000). Interestingly, the upward bending from linearity in the Pepperberg curve appeared at a reduced flash strength and rose more steeply. The basis was that elevated cGMP supported a greater fraction of open CNG channels and a greater influx of Ca^2+^. As a result, the content of Ca^2+^-bound recoverin was higher and a greater proportion of RK was not available to initiate shutoff of R^*^ until there was a sufficient light-induced fall in Ca^2+^. Thus, even though PDE levels were reduced, each R^*^ was able to generate more T^*^--E than normal. Reduced expression of PDE along with an increased number of PDE^*^ per R^*^ caused the Pepperberg curve to fully activate all PDE and approach the plateau with less intense flashes than for WT rods. Consistent with this explanation, the upward bending occurred with more intense flashes in mutant mice lacking recoverin (Makino et al., 2004) and occurred with less intense flashes in mutant mice expressing lower levels of RK (see below).

Under these conditions, the model was used to interrogate whether the upward bend reached its maximal rate of rise of 9–10 s, the time taken for the response recovery in the absence of RGS9 complex (Chen et al., 2000; Krispel et al., 2003a; Keresztes et al., 2004). The maximal slope within the range of the upward bend (convexity) was nearly 4 s, indicating that the maximal rate of rise was not achieved. Since the upward curvature rises less steeply for WT rods, we predict that the ratio of expression of RGS9 to PDE is adequate to ensure that T^*^--E shutoff would never rely on the T^*^ GTPase activity free from RGS9 acceleration at any flash strength.A theoretical, tenfold increment in PDE levels had minor effects in the opposite direction (red trace, Figure 5): $T_{sat}$ was somewhat shorter at every flash strength, the upward bending and the plateau occurred at slightly greater Ln(Φ) values.

Experiments addressing the plateau in the Pepperberg plot are not yet available. Challenging technical issues and slower mechanisms of light adaptation arise with the recording of responses that remain in saturation for such extended periods to extremely bright flashes, but in principle, it could be done. An exciting recent development is that a mutant mouse has been generated with greatly reduced expression of PDE (Morshedian et al., 2022), that could be useful for testing the model predictions.

#### Effects of changing the rate of R* phosphorylation

**Figure 6:**
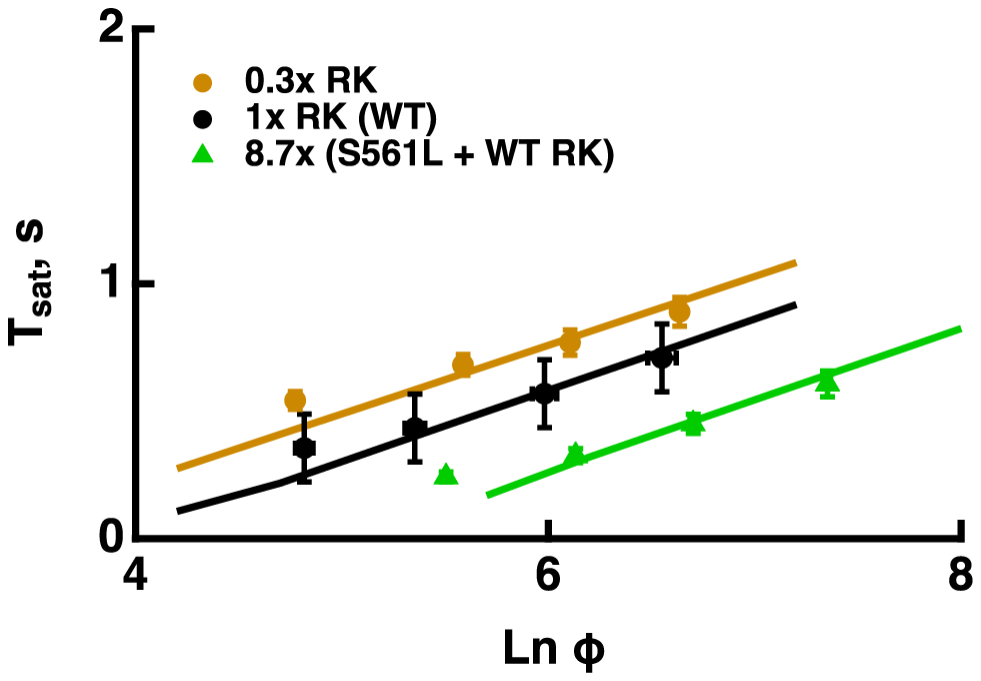
Changes in the rate of R^*^ shutoff affected *T*_*sat*_ without a change in τ_D_. Pepperberg plots for RK+/− rods with lower than normal levels of RK (0.3x RK), WT (1x RK) and rods expressing an excess of mutant, S561L RK as well as WT RK yielding a total RK content that was 8.7-fold higher than normal (S561L RK + WT). For modeling, the phosphorylation rate of R* was decreased 3-fold for RK+/− rods and increased 3-fold for S561L rods. *T*_*sat*_ was measured from mid-flash to 10% recovery. Adapted from Gross et al. (2012).

A reduction in RK expression slowed R^*^ shutoff, indicating that in large part, collision time between R^*^ and its kinase dominates the process. Hemizygous knockout of RK reduced RK levels by 70%, increased saturation times and shifted the X-intercept to lower flash strengths, however, it did not change the slope of the Pepperberg significantly (Figure 6, see also Sakurai et al., 2011; Gross et al., 2012). Upward bending in the RK+/− Pepperberg curve occurred at a lower flash strength than in WT rods (Sakurai et al., 2011), because slower R^*^ shutoff resulted in activation of more transducins, and a greater amount of T^*^--E that depleted more RGS9 at each flash strength.Overexpression of RK by two-fold or even four-fold had little effect on saturation behavior (Krispel et al., 2006; Sakurai et al., 2011). However, expression of a mutant S561L RK on a WT background reduced saturation time without a change in slope (Figure 6, see also Gross et al., 2012). The total level of both types of RK was 8.7-fold higher than normal and the mutant form contained a sequence that specified geranylgeranylation instead of farnesylation to enhance membrane affinity and quicken R^*^ phosphorylation. The constancy in slope in the face of changes in the rate of R^*^ shutoff helped to advance the argument that the rate-limiting step in flash response recovery was the shutoff of T^*^--E.

#### Effects of reducing Ca^2+^-dependent ROS-GC activity

**Figure 7:**
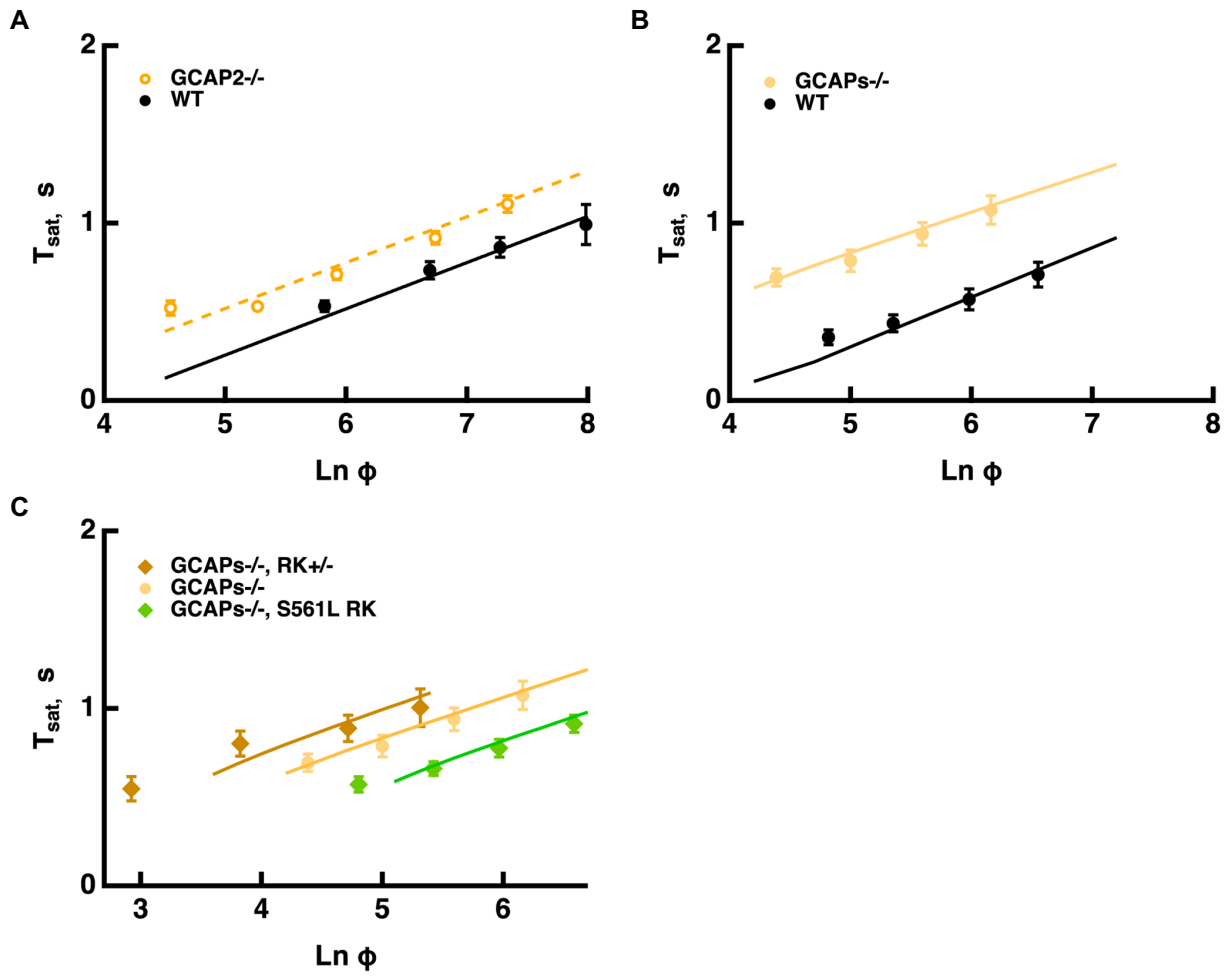
Longer *T*_*sat*_ without a change in slope of the Pepperberg relation upon reduction of Ca^2+^-dependent ROS-GC activity. **(A)** Pepperberg plots for GCAP2 knockout and wild type rods. Results were from Makino et al. (2008); *T*_*sat*_ was measured from mid-flash to 20% recovery. **(B)** Pepperberg plots for GCAPs knockout and wild type rods. Results were from Gross et al. (2012); *T*_*sat*_ was measured from mid-flash to 10% recovery. **(C)** Pepperberg plots for mutant RK rods from Figure 6 but on a GCAPs knockout background. For the parameter set used, the model did not yield saturating responses for the double mutants at the lowest *Ln* (Φ) values used experimentally. Results were from Gross et al. (2012); *T*_*sat*_ was measured from mid-flash to 10% recovery.

Mouse rods normally express two GCAPs that differ in their K_1/2_ for Ca^2+^: 46–47 nM for GCAP1 and 133 nM for GCAP2 (Makino et al., 2008, 2012). According to the model, $E_{char}^{*}$ changes with ROS-GC activity because the balance between cGMP synthesis and hydrolysis by E^*^ sets the cGMP levels and thereby affects response saturation. Knockout of both GCAPs removes Ca^2+^ feedback onto ROS-GC and increases the size of the single photon response five-to six-fold by allowing for a greater drop in cGMP after the photoisomerizations (Mendez et al., 2001; Burns et al., 2002; Chen et al., 2010; Makino et al., 2012). The model captured the increases in saturation times without a change in slope in the Pepperberg relation, because $\tau_{E^{*}}$ remained the same (Figure 7B). Because the effect of T^*^--E was opposed by a lower ROS-GC activity, Φo shifts to less intense flashes. Similarly, knockout of both GCAPs from mice expressing varying levels of RK had the same effect of increasing saturation time without a change in slope (Figure 7C). Importantly, the proportional increase in saturation time was similar for all three types of rods (*cf.* Figure 6).

Knockout of GCAP2 has a less dramatic effect as the maximal rate of cGMP synthesis at low Ca^2+^ drops to 40% and the overall K_1/2_ for Ca^2+^ shifts from 133 nM to ~47 nM (Makino et al., 2008). The slope of the Pepperberg is unchanged, but the X-intercept shifts to a lower value because of the response prolongation due to the reduction in Ca^2+^-dependent ROS-GC activity (Figure 7A).

#### Effects of removing CNG channel modulation by calmodulin

**Figure 8:**
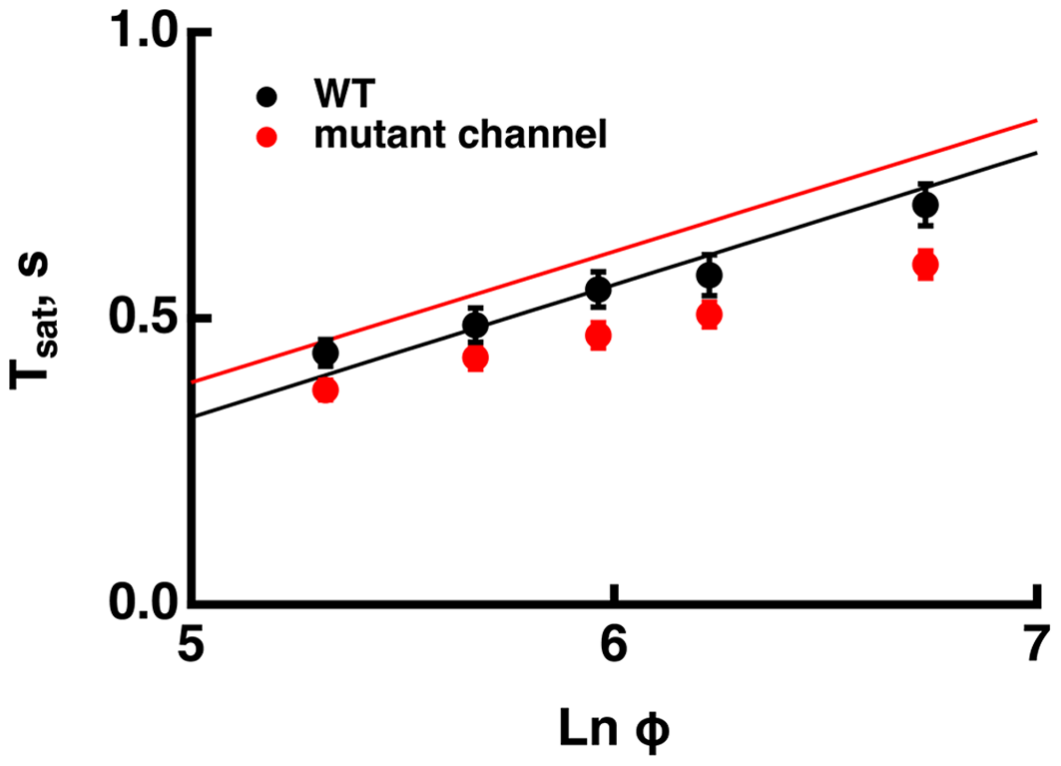
Increased CNG channel affinity for cGMP reduces saturation time with little change in Pepperberg slope in conflict with the model. *T*_*sat*_ was measured from flash onset to 25% recovery. The mutant CNG channel lacked calmodulin binding so for modeling, the K_cG_ was fixed at the minimum value at low Ca^2+^ of 13 μM. For the WT, the maximum value of 32 μM was used for high Ca^2+^. The Pepperberg relation predicted by the model for rods expressing the mutant channel was shifted to longer saturation times, in comparison to that of wild type rods. Adapted from Chen et al. (2010).

Mutant mouse rods expressing a mutant CNG channel that lacks the calmodulin binding site have reduced saturation times and a subtle but significant decrease in slope, τ_D_, with no change in dark current, sensitivity or dim flash response kinetics (Chen et al., 2010). These experimental findings were not predicted by the model (Figure 8). Ca^2+^/calmodulin lowers CNG channel affinity for cGMP in darkness so in the absence of calmodulin modulation, channel affinity for cGMP increases and dark current increases somewhat. During the response to a saturating flash, Ca^2+^ drops to a minimum so calmodulin no longer reduces the apparent affinity of the channel for cGMP, hence WT and mutant channels would reopen at the same concentration of cGMP. As cGMP levels recover and outer segment levels of Ca^2+^ rise, fewer WT channels will open compared to mutant channels. The steeper trajectory of the mutant rod response recovery would tend to shorten $T_{sat}$, but because the saturating response amplitude is larger in mutant rods, measurement of $T_{sat}$ to 20% recovery would be made after a longer delay. The net result in simulations was a slight extension of $T_{sat}$ at all flash strengths with the loss of CNG channel regulation by calmodulin, leaving the slope of the Pepperberg relation unchanged. The discrepancy between the experimental results and the predictions of the model indicate that other factors must come into play. The absence of calmodulin binding might indirectly affect regulation of the CNG channel in other ways, such as by affecting its phosphorylation state or by altering its interaction with Grb14. Knockout of Grb14 has the effect of reducing $T_{sat}$ and τ_D_ (Woodruff et al., 2014). Another possibility is that levels of free calmodulin in the outer segment might be changed and impact other targets such as CaM kinase.

## Summary and conclusion

After refining our mathematical model for phototransduction to incorporate: two Ca^2+^-dependent ROS-GC activities to reflect the expression of GCAP1 and GCAP2 in mouse rods, Ca^2+^-dependent shutoff of R^*^, Ca^2+^-dependence of the CNG channel affinity for cGMP, and regulation of T^*^--E shutoff by RGS9, it was applied to the analysis of Pepperberg plots of mutant and WT mouse rods. Our model differed somewhat in the treatment of disk surface cascade reactions and Ca^2+^-dependent regulations and in parameter choices, from other GWS models (e.g., Burns and Pugh, 2009; Chen et al., 2012; Invergo et al., 2014; Reingruber et al., 2020), so there were some minor quantitative differences, but all were in general agreement over their descriptions of the flash responses of WT mouse rods and those of mutant mice with various expression levels of GRK and RGS9. A distinct feature of our model is the inclusion of formalism describing RGS9 and PDE dynamics, that enabled us to explain three domains of Pepperberg plots: a linear segment, a segment with upward curvature, and a plateau. Other adjustments were made to explain the Pepperberg plots of various types of mutant mouse rods.

**Figure 9:**
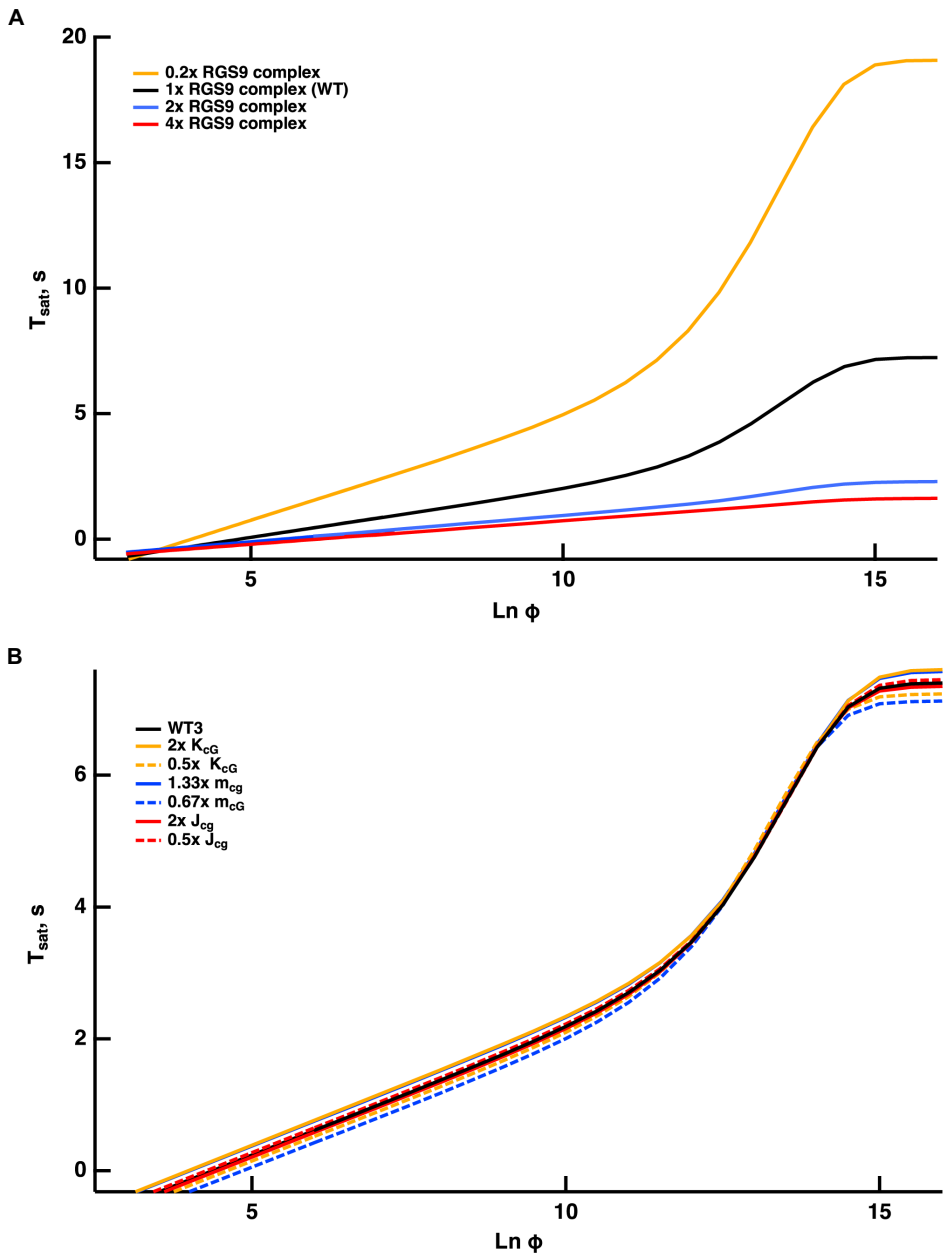
Model predictions for Pepperberg relations upon changing T*--E shutoff **(A)** or changing rod sensitivity **(B)**. Curves were generated for *T*_*sat*_ measured from mid-flash to 20% recovery. **(A)** Flash strength dependent deviations from linearity given by the model for various levels of RGS9 complex. **(B)** Shifts in the X-intercept predicted by the model for changes in sensitivity arising from alterations in the affinity of the CNG channel for cGMP or its cooperativity, or a change in dark current.

For flashes that send the response into saturation for brief periods, the time in saturation increases linearly with the natural logarithm of the flash strength. The slope of this linear domain gives the time constant of the cascade reaction that is rate-limiting to the flash response recovery. In WT rods, that reaction is the shutoff of T^*^--E (Krispel et al., 2006). Changes in the expression level of RGS9 complex affect the rate of T^*^--E shutoff by changing the delay before a diffusional encounter of T^*^--E with an RGS9 complex. In contrast, the shutoff of R^*^ is faster and changes in expression level of RK over a wide range, do not change the slope. After setting up our mathematical model to incorporate these features, the model explained many features of Pepperberg plots of various mutant rods and made some predictions of how response saturation would be affected by certain conditions.The Pepperberg relation for nearly every mutant rod that has been studied deviates from linearity at higher flash strengths, transitioning to an upward curvature, indicating that some condition has changed (e.g., Figures 1, 3). We successfully modeled the upward curvature as a flash-dependent decrease in the availability of RGS9 complex for accelerating T^*^--E shutoff (summarized in Figure 9A). As the number of photoisomerizations reaches critical levels, a stoichiometric depletion of RGS9 complex by an excess of PDE activation would force some T^*^--E to either wait their turn for RGS9 availability or to shut off without RGS9 complex. The flash strength at which the linear region transitions to upward curvature would then depend on how many transducins get activated per R^*^ and on the level of expression of RGS9 complex. Experimental observations verify both conditions (Figure 3). The model predicted that upward curvature would never asymptote to ~9 s, the time required for T^*^--E shutoff in the absence of RGS9 complex (Chen et al., 2000; Krispel et al., 2003a; Keresztes et al., 2004), because at extreme flash strengths, the Pepperberg relation would plateau upon activation of every PDE (Figures 5, 9A). It might at first seem paradoxical that the transition to upward bending would shift to fewer photoisomerizations with a drop in the expression level of PDE (Figures 5, 9A). However, the lower basal PDE activity permits higher cGMP levels at rest, more CNG channels open and higher internal Ca^2+^. Recoverin then binds a greater fraction of RK, slowing R^*^ shutoff and allowing activation of a greater number of T^*^. In addition, the greater dark current has a consequence that measurement of $T_{sat}$ after a criterion recovery would be made at a later time after the flash, an effect that would artifactually inflate $T_{sat}$. 

The Pepperberg relation also deviates from linearity for very short $T_{sat}$values because a finite time is required for Ca^2+^ levels to drop low enough to maximally accelerate ROS-GC activity. However, extrapolating the linear domain to the X-intercept yields Φo, the number of isomerizations that would just saturate the rod response with maximal ROS-GC activity and therefore stands as a gauge of sensitivity. Since flash sensitivity was unchanged by the level of RGS9 complex expression in mutant rods over a range varying from 0.3-fold to four-fold, the model predicted convergence of the Pepperberg relations of these rods, in rough agreement with our analyses of the experimental results (Figure 3). On the other hand, factors that influence flash sensitivity: Ca^2+^-dependent ROS-GC activity (Figure 7), the affinity and the cooperativity (Hill coefficient) of the channel for cGMP (Figure 8) do alter $T_{sat}$ and shift Φo without a change in slope of the Pepperberg relation (summarized in Figure 9B). Although moderate changes in the rate of R^*^ shutoff do not greatly affect flash sensitivity because of compensatory changes in Ca^2+^-dependent ROS-GC activity (Krispel et al., 2006; Sakurai et al., 2011; Gross et al., 2012), they nonetheless shift the X-intercept because bright flash responses operate in a regime where the Ca^2+^-dependent ROS-GC activity has reached a constant, maximal level (Figure 6). When Ca^2+^-dependent ROS-GC activity is absent, then changes in flash sensitivity do accompany the shifts in X-intercept (Gross et al., 2012).

For practical reasons, $T_{sat}$ is measured in experimental studies after some criterion recovery of the saturating response. This procedure inflates $T_{sat}$ values at each flash strength (Figure 2). Although it does not affect the slope of the Pepperberg relation, it introduces a left-shift in the X-intercept, thereby underestimating the true Φo. For a criterion recovery of 20%, a correction factor of 7.4-fold would improve the estimate of Φo. A larger correction factor would be needed for use of a criterion recovery >20%.

Increases in the circulating current, for example by a theoretical increase in the density of channels in the plasma membrane or by increasing single channel conductance, were predicted to have relatively minor effects on the Pepperberg relation (summarized in Figure 9B). Mutant mice with a change in CNG channel Hill exponent or channel density in the membrane are not yet available for comparison to model predictions.

In the current version of our model, $T_{sat}$ values reached a maximum with $Ln\left(\Phi \right)$ > 15, i.e., with flashes that activate every PDE (Figure 9). But larger $T_{sat}$ values are expected, based on the behavior of frog rods stimulated with exceedingly high flash strengths, because various phosphorylated states of R^*^, late stage photointermediates and opsin continue to activate the cascade until rhodopsin is regenerated to complete dark adaptation (Firsov et al., 2005). These factors were not incorporated into our model, and new experimental results are needed for mouse rods to fully understand the how the form of the Pepperberg plot changes as this flash regime is approached.

Modeling efforts by others (e.g., Chen et al., 2010; Invergo et al., 2014) have made it clear that not all features of light adaptation can be explained by known, Ca^2+^-dependent mechanisms regulating R^*^ lifetime, ROS-GC activity, and the affinity of the CNG channel for cGMP. Furthermore, additional mechanisms develop over more prolonged time courses (e.g., Calvert and Makino, 2002; Calvert et al., 2002; Krispel et al., 2003b). A future goal will be to incorporate additional modules for new mechanisms regulating the cascade into our model in order to understand the full extent of light adaptation.

## Data availability statement

Publicly available datasets were analyzed in this study.

## Author contributions

GC, PB, DA, and ED: conceptualization. GC, CK, HH, VG, PB, DA, ED, and CM: investigation. GC, PB, DA, ED, and CM: writing - original draft. GC, CK, HH, VG, PB, DA, and CM: review and editing. All authors contributed to the article and with the exception of ED, all approved the submitted version.

## Funding

This research was funded by NEI EY031702 (CM), EY011500 (VG), and NSF DMS1812601 (ED and HH). The contents are solely those of the authors and do not necessarily express the official views of the National Science Foundation nor those of the National Institutes of Health.

## Conflict of interest

The authors declare that the research was conducted in the absence of any commercial or financial relationships that could be construed as a potential conflict of interest.

## Publisher’s note

All claims expressed in this article are solely those of the authors and do not necessarily represent those of their affiliated organizations, or those of the publisher, the editors and the reviewers. Any product that may be evaluated in this article, or claim that may be made by its manufacturer, is not guaranteed or endorsed by the publisher.
