# Supplementary material for: Pepperberg plot: Modeling flash response saturation in retinal rods of mouse
Source: Front Mol Neurosci. 2023 Jan 13;15:1054449. doi: 10.3389/fnmol.2022.1054449 (PMC9880052; doi:10.3389/fnmol.2022.1054449)
Supplement: Supplementary file 1 [file Table_1.pdf]

Supplementary Materials

Table S1. Parameter set for modeling the WT mouse rod of Krispel et al. (2006).

Parameter	Units	Description	Value
		Outer Segment Geometry	
R	µm	Rod radius	0.685
H	µm	Rod height	23.6
ε_0	nm	Discal thickness	14.5
ν	-	Ratio between discal and interdiscal space	1
		Multistep R* Phosphorylation	
n_{step}	#	Number of phosphorylation states (including zero phosphorylations)	7
λ_0	s^{-1}	Maximum rate of phosphorylation	63
λ_j	s^{-1}	Rate of phosphorylation when in j^{th} state with j-1 phosphates	$\lambda_0*(n_{step}-j)/6$
μ_{max}	s^{-1}	Maximum rate of arrestin binding	60
μ_j	s^{-1}	Rate of arrestin binding for R* in j^{th} state with j-1 phosphates	$\mu_0*1_{[j>=4]}$
K_1	µM	Constant for recoverin binding in eq (7a)	4.5
K_2	µM	Constant for recoverin binding in eq (7a)	230
K_3	µM	Constant for recoverin binding in eq (7a)	3.4
K_4	µM	Constant for recoverin binding in eq (7a)	3.4
M	µM	Constant for recoverin binding in eq (7a)	6000
$[Rec]_{tot}$	µM	Total recoverin concentration	34
$[RK]_{tot}$	µM	Total rhodopsin kinase concentration	7
		Discal Cascade	
ν_0	#	Number of T* activated by unphosphorylated R*	330
k_ν	-	Exponential decay constant for phosphorylated R* affinity to activate T*	0.5
ν_j	#	Number of T* activated by R* in j^{th} phosphorylation state	$\nu_0*\exp(-k_\nu*(j-1))$

k_{T^*E}	$\mu m^2/s$	Coupling coefficient for T* activating E*	0.00725
$[T]_0$	μm^{-2}	Surface density of transducin on a disc	2500
$[PDE]_0$	μm^{-2}	Surface density of PDE on a disc	750
$[RGS9]_0$	μm^{-2}	Surface density of RGS9 on a disc	100
k_{cat}	s^{-1}	Hydrolysis rate of GTP by T*— —E complexed with RGS9	5.87
k_f	$\mu m^2/s$	Coupling coefficient for RGS9 binding T*— —E	3.68E-06
k_b	s^{-1}	Dissociation rate for RGS9 complexed with T*— —E	13.8
k_{hyd}	$\mu m^3/s$	Dark hydrolysis rate of cGMP by dark-activated PDE	2.80E-05
$k_{\sigma,hyd}$	$\mu m^3/s$	Light hydrolysis rate of cGMP by light-activated PDE	0.9
		<u>Synthesis of cGMP by Membrane Guanylate Cyclases</u>	
α_{max}	$\mu M/s$	Maximum rate of cGMP synthesis by ROS-GCs	50
α_{min}	$\mu M/s$	Minimum rate of cGMP synthesis by ROS-GCs	1
β	-	Proportion of ROS-GC activity due to GC1	0.4
K_{cyc1}	μM	Half-maximal concentration of $[Ca^{2+}]$ for GC1 activity	0.133
m_{cyc1}	-	Hill coefficient for GC1 activity	2.1
K_{cyc2}	μM	Half-maximal concentration of $[Ca^{2+}]$ for GC2 activity	0.047
m_{cyc2}	-	Hill coefficient for GC2 activity	1.9
		<u>Outer Segment Membrane Currents</u>	
j^{max}_{cG}	pA	Maximum CNG channel current	3550
f_{Ca}	-	Fraction of current through CNG channel carried by Ca^{2+}	0.06
m_{cG}	-	Hill coefficient of CNG channel for cGMP	3.5
$K_{cG,max}$	μM	Maximum value for half-maximal [cGMP] for CNG channel opening at high $[Ca^{2+}]$	32
$K_{cG,min}$	μM	Minimum value for half-maximal [cGMP] for CNG channel opening at low $[Ca^{2+}]$	13
K_{CaM}	μM	Half-maximal concentration of $[Ca^{2+}]$ for calmodulin modulation of CNG channel affinity for [cGMP]	0.06
m_{CaM}	-	Hill coefficient for calmodulin modulated CNG channel affinity for cGMP	2
j^{sat}_{ex}	pA	Maximum exchanger current	1.8
K_{ex}	μM	Half-maximal concentration of $[Ca^{2+}]$ for exchanger current	1.6

Minor adjustments were made for WT rods of other studies. Changes to the cascade for mutant rods were made as described in the text.
